# New Markers for the Assessment of Microvascular Complications in Patients with Metabolic Syndrome

**DOI:** 10.3390/metabo15030184

**Published:** 2025-03-10

**Authors:** Diana Nikolova, Zdravko Kamenov

**Affiliations:** Department of Internal Medicine, Aleksandrovska University Hospital, Medical University of Sofia, 1431 Sofia, Bulgaria; nikolowa.diana@abv.bg

**Keywords:** metabolic syndrome, type 2 diabetes mellitus, microvascular complications, biomarkers

## Abstract

**Background**: Metabolic syndrome is a complex disorder characterized by the coexistence of multiple risk factors, including dysglycemia, hypertension, dyslipidemia, and visceral obesity. Both metabolic syndrome and diabetes mellitus are closely associated with the onset of microvascular complications such as retinopathy, polyneuropathy, and nephropathy. **Methods**: This narrative review analyzed 137 studies published up to 2025, retrieved from PubMed and Crossref databases. The objective was to identify and evaluate potential biomarkers that could facilitate the early detection of microvascular complications in patients with metabolic syndrome. **Results**: Several biomarkers demonstrated a strong correlation with microvascular complications in individuals with metabolic syndrome. These findings suggest their potential role in early diagnosis and risk assessment. **Conclusions**: The identification of reliable biomarkers may enhance early detection and targeted interventions for microvascular complications in metabolic syndrome. Further research is essential to validate these markers and establish their clinical applicability in routine medical practice.

## 1. Introduction

### 1.1. Definition

The concept of metabolic syndrome (MetS) was first introduced in the 1920s when a Swedish physician demonstrated the link between high blood pressure, gout, and elevated blood sugar levels [1]. The World Health Organization (WHO) formally defined MetS in 1998, highlighting insulin resistance (IR) as a central component, particularly in individuals with type 2 diabetes or impaired glucose tolerance [2]. In contrast, the 2001 guidelines from the US National Cholesterol Education Program Adult Treatment Panel III (NCEP ATP III) redefined the criteria, excluding insulin resistance as a required diagnostic component [3].

Various definitions of metabolic syndrome exist. The WHO criteria published in 1999 required the presence of dysglycemia, which comprises impaired glucose tolerance, impaired fasting glucose or insulin resistance, and two of the following conditions: central obesity—waist-to-hip ratio > 0.90 (men), >0.85 (women) or body mass index > 30 kg/m^2^; dyslipidemia—triglycerides (TG): ≥1.695 mmol/L and high-density lipoprotein cholesterol (HDL-C) ≤ 0.9 mmol/L (men), ≤1.0 mmol/L (women); blood pressure: ≥140/90 mmHg; microalbuminuria: urinary albumin excretion ratio ≥ 20 μg/min or albumin–creatinine ratio ≥ 30 mg/g [4].

The NCEP definition includes the presence of at least three of the following: central obesity—waist circumference ≥ 102 cm for men, ≥88 cm for women; dyslipidemia—TG ≥ 1.7 mmol/L; HDL-C < 1.03 mmol/L for men, <1.29 mmol/L for women; blood pressure (BP) ≥ 130/85 mmHg; fasting plasma glucose ≥ 6.1 mmol/L [5].

In 2006, the International Diabetes Federation (IDF) introduced ethnicity-specific waist circumference cutoffs for diagnosing central obesity, reinforcing its role as a fundamental criterion for MetS. The definition of the syndrome required the presence of central obesity, defined as waist circumference with ethnicity-specific values and two of the following: elevated triglycerides: >1.7 mmol/L or treatment for this; reduced HDL cholesterol: <1.03 mmol/L in men, <1.29 mmol/L in women or taking specific treatment for it; elevated blood pressure: systolic BP > 130 or diastolic BP > 85 mm Hg or undergoing antihypertensive therapy; elevated fasting plasma glucose (FPG): >(5.6 mmol/L) or previously diagnosed type 2 diabetes. If BMI is >30 kg/m^2^, central obesity can be assumed, and waist circumference does not need to be measured [6].

The American Diabetes Association (ADA) updated the NCEP ATP III criteria, with this definition requiring waist circumference in men ≥ 102 cm, in women ≥ 88 cm; triglycerides ≥ 1.7 mmol/L; HDL cholesterol of in men < 1.03 mmol/L, in women < 1.29 mmol/L; blood pressure ≥ 130/85 mm Hg or undergoing antihypertensive therapy; elevated fasting glucose ≥ 5.6 mmol/L or taking a drug for hyperglycemia [7]. Metabolic syndrome is currently diagnosed when at least three of the five above-mentioned factors are present [8].

### 1.2. Epidemiology

The prevalence of the metabolic syndrome varies widely, ranging from 10% to 84% depending on factors such as diagnostic criteria, age, sex, ethnicity, and geographic region [1]. It is more common in individuals over 60 years old, with ethnic disparities in its distribution. For instance, women are disproportionately affected among Mexican American and African American populations, while prevalence rates among Caucasians remain relatively equal between sexes.

In Europe, approximately 25% of the population is affected by MetS [9], whereas in North America, particularly the United States, prevalence is estimated at around 35%, with similar trends observed in Brazil [10]. In Asia, the overall prevalence mirrors that of Europe, but significant ethnic variations exist. For example, approximately, the metabolic syndrome affects 35% of the Korean population, whereas only 6% of Tibetans meet the criteria [11,12].

The metabolic syndrome is a significant risk factor for cardiovascular disease [13], doubling the likelihood of its development within a decade. Additionally, individuals with MetS face a substantially increased risk—up to 4.1 times—of progressing to type 2 diabetes [14]. The study by Shoaib Asghar et al. showed that approximately 65% of MetS patients also have type 2 diabetes mellitus (T2DM) [15], while the remaining population is five times more likely to develop the condition [8]. Given this strong association, a significant proportion of individuals with metabolic syndrome are expected to experience microvascular complications, further emphasizing the need for early detection and management.

### 1.3. Pathophysiology

The development of the metabolic syndrome is driven by a complex interplay between the genetic predisposition and environmental factors [10]. Several key risk factors contribute to its onset, including age, ethnicity, obesity, a family history of diabetes, and comorbid conditions such as polycystic ovary syndrome, non-alcoholic fatty liver disease, sleep apnea, and cardiovascular disease [16]. Although MetS is commonly linked to obesity, studies have shown that it can also occur in non-obese individuals, particularly among smokers and former smokers living in rural areas [17].

The pathophysiology of MetS involves multiple interrelated mechanisms, with insulin resistance, chronic low-grade inflammation, and neurohormonal dysregulation playing central roles in its progression and eventual transition to type 2 diabetes mellitus [18]. Among these, abdominal obesity serves as a primary driver, exacerbating metabolic dysfunction through the increased release of inflammatory mediators and free fatty acids (FFAs) [19].

Under normal conditions, pancreatic β-cells respond to elevated blood glucose levels by secreting insulin, which facilitates glucose uptake, suppresses hepatic gluconeogenesis, and promotes glycogen synthesis. Insulin also plays a crucial role in lipid metabolism by inhibiting lipolysis [20]. However, in insulin-resistant states, these regulatory processes become impaired, resulting in elevated circulating FFAs, dysregulated glucose transport, and enhanced gluconeogenesis [21]. Additionally, insulin resistance disrupts vascular homeostasis by promoting vasoconstriction and increasing blood pressure, thus contributing to a heightened risk of cardiovascular disease [9].

At the molecular level, insulin exerts its effects through the activation of the phosphoinositide 3-kinase (PI3K) and mitogen-activated protein (MAP) kinase signaling pathways. While the PI3K-Akt pathway enhances endothelial nitric oxide synthase (eNOS) activity, promoting vasodilation and glucose uptake via the GLUT 4 transporter, the MAP kinase pathway stimulates endothelin-1 production, which induces vasoconstriction. Insulin resistance disrupts the PI3K-Akt pathway, impairing GLUT 4 translocation and reducing nitric oxide bioavailability [22]. This results in endothelial dysfunction, increased oxidative stress due to reactive oxygen species (ROS), and the development of hypertension [23]. Simultaneously, FFAs contribute to dyslipidemia by promoting very low-density lipoprotein (VLDL) synthesis and reducing high-density lipoprotein (HDL) cholesterol levels [24].

Chronic low-grade inflammation is another hallmark of MetS. Elevated levels of pro-inflammatory cytokines such as interleukin-6 (IL-6), C-reactive protein (CRP), and tumor necrosis factor-α (TNF-α) are associated with insulin resistance by interfering with insulin receptor signaling [9,18]. Reduced adiponectin secretion in individuals with visceral obesity further exacerbates metabolic dysfunction [25]. TNF-α has been implicated in lipolysis induction, increased FFA levels, and the suppression of adiponectin production [26], while IL-6 promotes CRP synthesis, reinforcing systemic inflammation and a prothrombotic state [27].

The innate immune system, particularly Toll-like receptors (TLRs), also plays a significant role in metabolic dysregulation. TLR4 activation, commonly observed in obesity, triggers pro-inflammatory signaling cascades, sustaining chronic inflammation and further impairing insulin sensitivity. This further contributes to the pathogenesis of MetS [28,29]. The renin–angiotensin system (RAS) becomes excessively activated in individuals with obesity and insulin resistance, leading to increased production of angiotensin II (Ang II) [30]. This promotes ROS generation, the upregulation of lectin-like oxidized low-density lipoprotein receptor-1 (LOX-1), and endothelial dysfunction, thereby accelerating the progression of MetS and its associated complications [18].

### 1.4. Microvascular Complications

Complications arising from type 2 diabetes mellitus are broadly categorized into microvascular and macrovascular complications. Microvascular complications primarily affect small blood vessels and include diabetic retinopathy, diabetic nephropathy, and diabetic neuropathy [31]. These complications can manifest at the time of diabetes diagnosis and, in some cases, even in individuals with prediabetes, suggesting that some patients may have an increased predisposition to vascular damage in the presence of dysglycemia [32,33].

Diabetic retinopathy (DR) is one of the leading causes of vision impairment globally [34] and represents the most frequently observed microvascular complication in individuals with metabolic syndrome and T2DM [15]. Based on the presence or absence of retinal neovascularization, DR is classified into nonproliferative diabetic retinopathy (NPDR) and proliferative diabetic retinopathy (PDR) [35]. NPDR is associated with symptoms such as difficulties with night vision, blurred vision, and localized inflammation in retinal blood vessels. In PDR, impaired circulation results in inadequate oxygen delivery to the retina, leading to disease progression [36].

A key pathological feature in the early stages of DR is pericyte loss, which contributes to the formation of microaneurysms—often the earliest clinical sign of the condition [37]. Major risk factors for DR development include hyperglycemia, hypertension, nephropathy, obesity, dyslipidemia, and smoking [38]. Evidence from the Diabetes Prevention Program (DPP) indicates that 7.9% of individuals with impaired fasting glucose exhibit retinopathy [39]. Additionally, studies conducted in Chinese populations reveal that retinopathy is prevalent not only among patients with T2DM but also in those with metabolic syndrome [40,41]. While hyperglycemia is a primary driver of DR, other components of the metabolic syndrome, such as hypertension, dyslipidemia and obesity, contribute to oxidative stress and vascular damage, further exacerbating pericyte loss and disease progression [42].

According to a study by Asghar et al., diabetic nephropathy (DN) is the second most common microvascular complication in patients with metabolic syndrome and T2DM [15]. It is a major cause of end-stage renal disease (ESRD) among diabetic patients [43]. Several metabolic and hemodynamic factors characteristic of the metabolic syndrome—such as dyslipidemia, insulin resistance, hyperglycemia, hypertension, and activation of the reninangiotensin system—play crucial roles in its pathogenesis. Visceral obesity has been identified as a key driver of these abnormalities [44].

Chronic inflammation, oxidative stress, and excessive ROS production contribute to mesangial cell hypertrophy, which ultimately leads to renal dysfunction [45]. Clinically, DN is characterized by a triad of persistent albuminuria, progressive decline in glomerular filtration rate (GFR), and hypertension [46]. Given that microalbuminuria is often the earliest detectable abnormality, it is a critical parameter for screening and early diagnosis of diabetic nephropathy [47].

The American Diabetes Association categorizes diabetic neuropathies into diffuse neuropathy, mononeuropathies, and radiculopathies or polyradiculopathies. Within diffuse neuropathy, two subtypes are recognized: distal symmetric polyneuropathy and autonomic neuropathy [48]. The development of diabetic polyneuropathy (DPN) is multifactorial, with growing evidence suggesting that its underlying mechanisms may differ between type 1 and type 2 diabetes. In T2DM, major contributors to DPN include oxidative stress, vascular dysfunction, and metabolic disturbances. Primary risk factors for DPN development include hyperglycemia, the duration of diabetes, and hypertension, while obesity and smoking have also been identified as contributing factors [49]. An increasing number of studies highlight the link between MetS, prediabetes, and obesity with DPN onset [50].

Diabetic autonomic neuropathy (DAN) is a serious and frequently underdiagnosed complication of diabetes, often remaining asymptomatic in its early stages [51]. Since the autonomic nervous system regulates various physiological functions, DAN can present with a wide range of clinical manifestations, including orthostatic hypotension, resting tachycardia, impaired hypoglycemia awareness, gastroparesis, erectile dysfunction, neurogenic bladder, and sudomotor dysfunction [44]. Several pathogenic mechanisms have been proposed for DAN, including polyol pathway activation with sorbitol accumulation, protein kinase C activation, oxidative stress, and endothelial dysfunction [52]. The ADA defines cardiac autonomic neuropathy (CAN) as a condition characterized by abnormal cardiovascular autonomic regulation in diabetes, after other potential causes have been ruled out [53]. Major risk factors for CAN in T2DM patients include age, sex, ethnicity, insulin resistance, and the presence of other microvascular complications such as nephropathy, retinopathy, and peripheral neuropathy [54,55]. Studies also suggest that CAN is frequently observed in individuals with MetS and impaired glucose tolerance, emphasizing the close association between autonomic dysfunction and metabolic abnormalities [56,57].

### 1.5. Biomarkers

#### 1.5.1. Angiopoetin-like Protein 4 and Angiopoetin-like Protein 8

**Figure 1:**
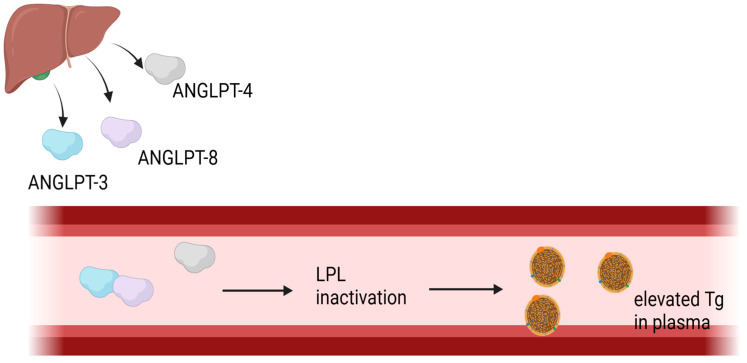
The role of ANGPTL-3, ANGPTL-4, and ANGPTL-8 in Tg metabolism.

Angiopoietin-like proteins (ANGPTLs) are a family of secreted glycoproteins consisting of eight members (ANGPTL1–8) [58]. Among them, ANGPTL3, ANGPTL4, and ANGPTL8 (Figure 1) function as potent inhibitors of lipoprotein lipase, significantly influencing lipid metabolism [59].

ANGPTL4, initially described in 2000 [60], plays a role in triglyceride metabolism by regulating lipoprotein lipase activity during fasting. It is primarily expressed in liver and adipose tissue [61], with additional expression in muscle under fasting conditions [59]. By hydrolyzing triglycerides in chylomicrons and VLDLs, ANGPTL4 contributes to the modulation of circulating lipid levels [61]. Beyond its metabolic functions, ANGPTL4 also has pro-inflammatory properties, influencing inflammatory pathways [62]. A study by Eman Al Shawaf et al. of 122 patients linked elevated ANGPTL4 levels with diabetic nephropathy, suggesting its potential as a predictive biomarker for microvascular complications [63]. Additionally, research has demonstrated that ANGPTL4 may contribute to diabetic retinopathy by increasing vascular permeability through the activation of hypoxia-inducible factor-1 in hypoxic retinal Müller cells [64,65,66].

ANGPTL8, originally identified as lipasin in 2012 [58], is predominantly produced in liver and white adipose tissue [67], with additional expression in the intestinal epithelium [68]. Unlike ANGPTL4, ANGPTL8 requires ANGPTL3 to exert its lipoprotein lipase-inhibitory function [59]. Insulin increases ANGPTL8 levels in liver and adipose tissue [69] and its upregulation in insulin-resistant states is associated with chronic inflammation, increased free fatty acids, and reduced adiponectin secretion [70]. Elevated ANGPTL8 levels have been found to correlate with fasting glucose, triglycerides, obesity, and metabolic syndrome [58]. Furthermore, studies suggest that ANGPTL8 may be a biomarker for diabetic retinopathy and diabetic nephropathy, with evidence supporting its correlation with serum creatinine levels and glomerular filtration rate (GFR) decline. A recent study by Hana Th. Al Majed et al. among the Arab population found a significant elevation of ANGPTL8 in nephropathy compared to T2DM patients. Additionally, the team found a positive correlation of ANGPTL8 with serum creatinine and a negative correlation with eGFR and urinary creatinine in people with nephropathy, making this adipokine a possible regulator in the development of nephropathy [71,72].

#### 1.5.2. Lipocalin-2/NGAL

Lipocalin-2 (LCN2), also known as neutrophil gelatinase-associated lipocalin (NGAL), has been proposed as a biomarker for renal dysfunction, MetS, and T2DM [73]. This 198-amino-acid adipocytokine [74] is secreted by neutrophils, macrophages, dendritic cells [75] osteoblasts, and adipocytes [73].

LCN2 exists in three distinct forms: a monomer (25 kDa) secreted by renal tubules, a homodimer (45 kDa) released during inflammation, and a 135 kDa complex bound to matrix metalloproteinase-9 (MMP-9) [75]. LCN2 is highly expressed in adipose tissue [76] with elevated levels observed in obese and insulin-resistant individuals [74]. There is evidence suggesting that LCN2 may play a protective role in insulin sensitivity, although its precise function remains under investigation [77].

**Figure 2:**
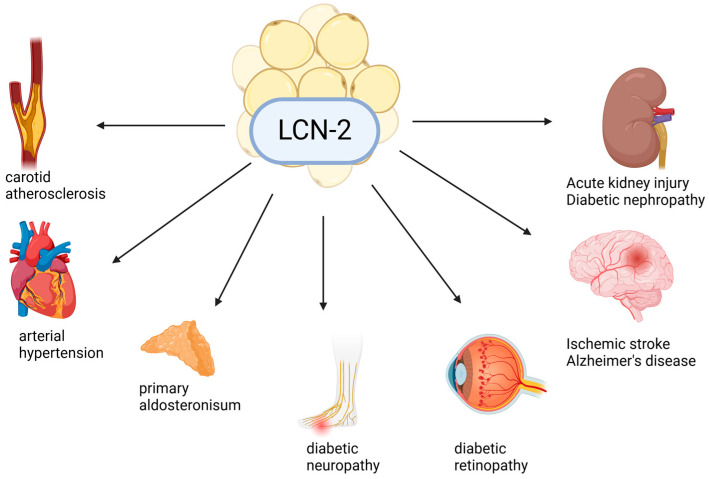
The association between lipocalin-2 and various medical conditions.

LCN2 expression is regulated by insulin through the phosphatidylinositol 3-kinase (PI3K) and mitogen-activated protein kinase (MAPK) pathways [74]. High levels of LCN2 are also associated with arterial hypertension, another component of MetS, with higher levels found in hypertensive patients compared to normotensive patients (Figure 2) [78]. Additionally, LCN2 has been proposed as a biomarker for primary aldosteronism, the most frequent cause of endocrine-related hypertension (Figure 2) [79]. Recent studies suggest that LCN2 is a reliable biomarker for microvascular complications, including diabetic retinopathy [80], nephropathy [81], and carotid atherosclerosis (Figure 2) [82].

By modulating iron homeostasis, LCN2 contributes to oxidative stress, which, in turn, enhances advanced glycation end-product (AGE) formation and increases vascular permeability via MMP-9 activation. Additionally, LCN2 is implicated in neurological disorders, such as Alzheimer’s disease and ischemic stroke (Figure 2), and has been proposed as an early biomarker for diabetic neuropathy (Figure 2). It is thought to contribute to DPN by promoting neuronal apoptosis and demyelination [83]. In addition to chronic conditions, LCN2 can also be associated with acute kidney injury (Figure 2) [84]. Furthermore, urinary NGAL levels have been investigated in a pediatric population, where it has been suggested as a useful marker for the risk of early kidney damage in obese children with insulin resistance, even in the absence of T2DM [85].

#### 1.5.3. CTRP

Adipokines affect glucose and fat metabolism, insulin sensitivity and inflammatory processes [86]. The C1q tumor necrosis factor-related protein (CTRPs) is a family of 15 adipokines (CTRP1–CTRP15) that play an important role in the regulation of endothelial dysfunction, chronic inflammation, glucose, and lipid mechanisms [87]. Although adipose tissue is the primary source of secretion, CTRPs are also expressed in liver and skeletal muscle [88].

**Figure 3:**
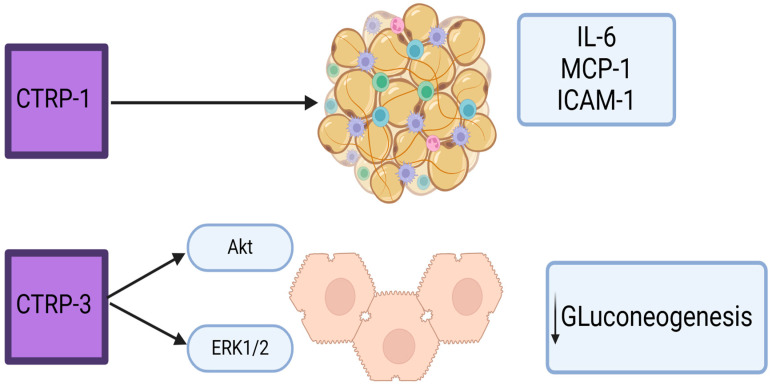
CTRPs role in inflammation and metabolism.

CTRP-1 is highly expressed in adipose tissue and plays a role in glucose homeostasis. It promotes AMP-activated kinase (AMPK) activation, thereby improving insulin resistance. Elevated CTRP-1 levels are observed in T2DM, metabolic syndrome, coronary artery disease, and hypertension [89]. It also influences inflammatory pathways by increasing the expression of IL-6, monocyte chemoattractant protein-1 (MCP-1), and intracellular adhesion molecule-1 (ICAM-1) (Figure 3) [90].

CTRP-3 has anti-inflammatory properties, reducing TNF-α and IL-6 levels [91]. Patients who have coronary artery disease exhibit reduced levels of CTRP3 [92]. It also inhibits hepatic gluconeogenesis through the Akt and ERK1/2 pathways (Figure 3) [93]. Studies have found elevated levels of CTRP-3 in patients with type 2 diabetes or prediabetes compared to patients with normal glucose tolerance [94]. On the other hand, low levels of CTRP-3 were found in patients with diabetic peripheral neuropathy [95].

CTRP-9 enhances AMPK, Akt, and MAPK signaling, leading to improved glucose uptake and vascular function [96]. It has been linked to diabetic retinopathy, nephropathy, and cardiac autonomic neuropathy. Additionally, CTRP-9 levels are higher in individuals with metabolic syndrome, obesity, and impaired fasting glucose. A study among 262 patients with type 2 diabetes mellitus has linked serum levels of CTRP-9 to cardiac autonomic neuropathy and peripheral neuropathy [97]. The protective function of CTRP-9 in the vascular endothelium has been demonstrated in animal experiments, showing its beneficial role in diabetic retinopathy and nephropathy [98,99]. In addition to microvascular complications, CTRP-9 levels are also elevated in patients with components of the metabolic syndrome such as obesity and impaired fasting glycemia [100].

#### 1.5.4. Apelin

Apelin is a regulatory peptide and adipokine widely distributed in various organs, including the nervous system, lungs, liver, cardiovascular system, kidneys, and gastrointestinal tract [101]. It exists in four active forms (apelin-13, apelin-17, apelin-36, and pyroglutaminated apelin-13) [102]. Apelin exerts its effects by binding to the APJ receptor, a G-protein-coupled receptor [103].

**Figure 4:**
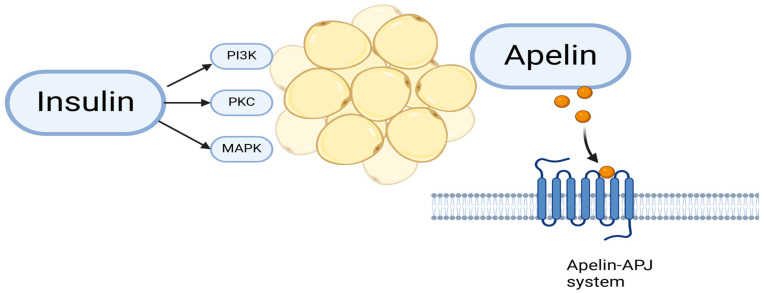
Apelin secretion and its receptor.

Apelin is secreted by adipocytes under the influence of insulin, enhancing glucose uptake and playing a role in insulin sensitivity regulation [104]. Insulin regulates the expression of apelin in adipocytes through the stimulation of phosphatidylinositol 3-kinase, protein kinase C and MAPK (Figure 4). Studies indicate that apelin expression declines during fasting and increases postprandial [105]. Elevated plasma apelin levels have been observed in obese individuals, patients with T2DM, and those with MetS [106,107]. In a Bulgarian population study of 99 men, Angelova et al. found elevated serum apelin levels in individuals with MetS compared to healthy controls [108].

When investigating the genetic role of the apelin-APJ system (Figure 4) in MetS among 1005 patients, significantly increased levels of apelin-36 were found in those with MetS [103]. Another study showed a positive association between apelin-12 and apelin-36 levels and insulin resistance [109]. Apelin has a beneficial effect on insulin sensitivity [110]. and improves dyslipidemia [101].

The apelin/APJ system is involved in vascular function and may exert protective effects in diabetic retinopathy by preventing pericyte loss and vascular leakage [111]. Through its effects on vascular integrity, apelin expression plays a protective role in the early stages of DR [112]. Elevated serum apelin levels have been detected in individuals with diabetic polyneuropathy, with improvements noted following glycemic control and neurotrophic therapy (e.g., alpha-lipoic acid and methylcobalamin). These findings suggest that apelin may serve as a biomarker for early detection and therapeutic monitoring of DPN [113].

#### 1.5.5. Galectin-3

Galectin-3 (Gal-3) is a 30 kDa protein, previously known as carbohydrate-binding protein-35 (CBP-35), that belongs to the family of β-galactoside-binding proteins [114,115]. It is expressed by various cells, including epithelial, immune, endothelial, and neuronal cells [116]. Galectin-3 is present intracellularly and extracellularly. It interacts with the extracellular matrix and cell surface glycoproteins using its carbohydrate-recognition domain, and with peptides via its N-terminus domain. It plays a role in cell adhesion, growth, differentiation, apoptosis, and angiogenesis [117]. Gal-3 is associated with the regulation of fibrosis, tumorigenesis, innate immune responses against pathogens, and immune suppression in tumors [118]. It is easily secreted to the cell surface and can be found in biological fluids, making it a highly sensitive biomarker for diagnosis and prognosis of various pathological conditions, including heart disease, kidney disease, diabetes mellitus, viral infections, autoimmune diseases, neurodegenerative diseases, and tumor formation [119].

A study using gene-modified mice found that Gal-3 inhibits insulin receptor signaling, which affects insulin action in adipocytes, muscle cells, and hepatocytes while promoting adipose tissue inflammation [120]. Circulating levels of Gal-3 are positively correlated with MetS components, including obesity, diabetes, dyslipidemia, and hypertension [121]. Yilmaz et al. identified Gal-3 as a significant predictor of diabetes in their study involving 118 participants [122].

**Figure 5:**
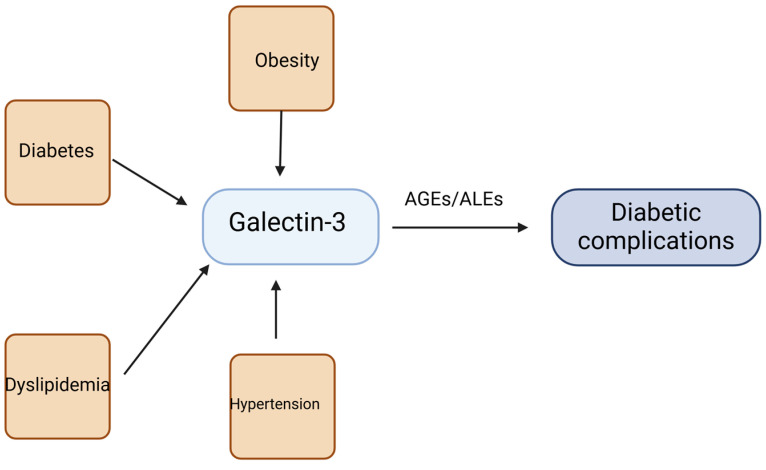
The role of galectin-3 in metabolic disorders and diabetic complication.

Galectin-3 plays a role in the development of diabetic complications by binding to AGEs and advanced lipoxidation end-products (ALEs) (Figure 5) [123]. A study conducted by Surendra Kumar et al. investigated the levels of Gal-3 among patients with T2DM and obesity, who also suffered from microvascular complications. The study found that patients with nephropathy exhibited significantly higher levels of serum galectin in those with macroalbuminuria, compared to those with microalbuminuria [115]. This finding is consistent with the results of similar studies conducted by Hodeib et al. [117] and Jin Qi-hui et al. [124]. In addition to nephropathy, high serum Gal-3 was significantly associated with the development of retinopathy and neuropathy [115,116].

### 1.6. Recent Advances in Diabetes Treatment: Mechanisms of Action of Novel Drugs

The process of glucose reabsorption in the kidneys occurs through specific glucose transport proteins, with sodium–glucose cotransporter 2 (SGLT2) being the most significant. This transport protein, found in the proximal tubule of the kidney, is responsible for the majority of renal glucose reabsorption, facilitating the movement of glucose back into the bloodstream after filtration through the glomeruli [125].

SGLT-2 inhibitors are a group of drugs primarily used to manage type 2 diabetes. They work by inhibiting the SGLT-2 protein. By blocking this protein, these inhibitors reduce the kidney’s ability to reabsorb glucose back into the bloodstream. As a result, excess glucose is excreted through urine, leading to lower blood glucose levels [126]. Meta-analyses of clinical trials have shown that SGLT2 inhibitors lead to significant weight reduction. This is largely attributed to energy loss through glucose excretion and a metabolic shift toward ketone and fatty acid utilization, which enhances fat breakdown and promotes weight loss [127]. SGLT2 inhibitors offer renoprotective benefits by correcting glomerular hyperfiltration, improving oxygenation, and reducing mitochondrial damage. These effects decrease oxidative stress, inflammation, and fibrosis, while enhancing tubule–glomerular feedback, ultimately preserving kidney function and slowing the progression of kidney disease [128]. Despite low cardiac SGLT2 expression, SGLT-2 inhibitors improve heart function by enhancing sodium handling, contractility, and energy efficiency while reducing oxidative stress. Beyond glucose control, they offer cardiovascular benefits, including anti-inflammatory and plaque-stabilizing effects [129]. Studies suggest that SGLT-2 inhibitors may be a promising treatment for non-alcoholic fatty liver disease (NAFLD) by regulating lipid metabolism, decreasing liver fat deposits, and protecting hepatocytes from apoptosis [130]

Glucagon-like peptide-1 (GLP-1), a hormone from the incretin family, is released after food ingestion and plays a key role in glucose regulation. Its beneficial effects for T2DM treatment include stimulating insulin secretion and suppressing glucagon release in a glucose-dependent manner and slowing gastric emptying [131].

GLP-1 receptor agonists (GLP-1 RAs) reduce appetite and promote satiety by acting on the central nervous system, leading to reduced calorie intake and weight loss. Their combined benefits in glucose regulation and weight management have made them a popular option not only for diabetes treatment but also for weight loss in non-diabetic individuals. A co-agonist targeting both the GLP-1 and gastric inhibitory polypeptide (GIP) receptors marks a significant breakthrough, demonstrating improved effectiveness in weight loss and expanding the range of available treatments for obesity.

Food intake is regulated through complex interactions between hormones, nutrients, and brain regions. GLP-1 receptor agonists enhance signals in the brainstem, promoting satiety and reducing appetite by activating serotonergic neurons and releasing glutamate. They also influence the hypothalamus, increasing anorexigenic peptides and reducing orexigenic peptides, leading to decreased food intake. In the mesolimbic reward pathway, GLP-1 reduces dopamine release, lowering the reward response to food. Additionally, GLP-1 RAs improve leptin sensitivity, enhancing appetite suppression [132]. Beyond glucose regulation, GLP-1 receptor agonists exhibit anti-inflammatory properties by modulating immune cell signaling, suppress pro-inflammatory cytokines via NF-κB inhibition, activate AMPK pathways, and reduce oxidative stress. They also influence lipid metabolism, promoting fat breakdown and reducing lipogenesis [133].

## 2. Discussion

This review highlights the potential role of several biomarkers—angiopoietin-like protein 4 (ANGPTL4), angiopoietin-like protein 8 (ANGPTL8), lipocalin-2 (LCN2/NGAL), CTRP-3, CTRP-9, apelin, and galectin-3 (Gal-3)—in the early detection of microvascular complications in individuals with MetS. These biomarkers demonstrate strong correlations with retinopathy, nephropathy, and neuropathy, suggesting their potential diagnostic and prognostic utility.

ANGPTL4 and ANGPTL8, which regulate lipid metabolism and inflammatory responses, have been associated with both diabetic nephropathy and retinopathy [63,64,65,66,71,72]. Elevated levels of ANGPTL4 contribute to vascular permeability, a hallmark of diabetic retinopathy [64,65,66], while ANGPTL8 has been linked to nephropathy progression [72]. Similarly, lipocalin-2 has emerged as a promising marker for nephropathy [81], retinopathy [80], and neuropathy, potentially due to its role in oxidative stress and inflammation [83].

CTRP-3 and CTRP-9, known for their metabolic regulatory functions [93] have been implicated in both microvascular and cardiovascular complications, with CTRP-9 showing significant associations with diabetic neuropathy and endothelial dysfunction [94,95,96,97,98,99]. Apelin, a regulator of insulin sensitivity [104], has demonstrated protective effects in diabetic retinopathy [111], while galectin-3 has been identified as a key factor in fibrosis and inflammatory responses [118], particularly in nephropathy and neuropathy [115,116,117].

The therapeutic potential of GLP-1 receptor agonists and SGLT2 extends beyond glucose regulation, as these drugs interact with biomarkers to provide anti-inflammatory, cardioprotective, and renoprotective effects. SGLT2 inhibitors may modulate ANGPTL4 expression, improving lipid profiles and reducing cardiovascular risk [134], while GLP-1 receptor agonists reduce lipocalin-2 levels, contributing to lower oxidative stress and improved renal health [135,136]. Moreover, apelin levels are increased by SGLT2 inhibitors, enhancing vascular function and improving cardiac outcomes [137].

While some studies support the clinical utility of these biomarkers, inconsistencies in the reported findings highlight the need for standardized methodologies. Differences in study populations, measurement techniques, and diagnostic criteria may contribute to variability in biomarker efficacy. Furthermore, most studies have been conducted in specific ethnic groups, limiting the generalizability of results.

## 3. Conclusions

Both diabetes mellitus and metabolic syndrome are closely linked to the onset of microvascular complications, including retinopathy, polyneuropathy, and nephropathy. This review suggests that specific biomarkers—such as angiopoietin-like protein 4, angiopoietin-like protein 8, lipocalin-2, CTRP-3, CTRP-9, apelin, and galectin-3—show a strong correlation with these complications in individuals with MetS. Their potential role in early diagnosis makes them promising candidates for identifying microvascular damage at an early stage, thereby facilitating timely intervention and reducing disease progression.

## 4. Future Directions

Further large-scale, multicenter studies are required to validate these findings and explore the combined predictive value of multiple biomarkers. Investigating the mechanistic pathways linking these markers to microvascular damage may also provide novel therapeutic targets. Additionally, integrating biomarker panels with advanced imaging and artificial intelligence-based prediction models could enhance early diagnosis and risk stratification in metabolic syndrome.

## Abbreviations

The following abbreviations are used in this manuscript:

MetS	Metabolic syndrome
WHO	World Health Organization
IR	Insulin resistance
NCEP	The 2001 US National Cholesterol Education Program Adult Treatment Panel III
TG	Triglycerides
HDL	High-density lipoprotein cholesterol
BP	Blood pressure
IDF	International Diabetes Association
FPG	Fasting plasma glucose
BMI	Body mass index
ADA	American Diabetes Association
T2DM	Type 2 diabetes mellitus
FFA	Free fatty acid
PI3K	Phosphoinositide 3-kinase
MAP	Mitogen-activated protein
eNOS	Endothelial nitric oxide synthase
ROS	Reactive oxygen species
VLDL	Very low-density lipoproteins
IL-6	Interleukin-6
CRP	C-reactive protein
TNF-α	Tumor necrosis factor alfa
TLRs	Toll-like receptors
RAS	Renin–angiotensinogen system
Ang II	Angiotensin II
LOX-1	Low-density lipoprotein receptor-1
DR	Diabetic retinopathy
NPDR	Nonproliferative diabetic retinopathy
PDR	Proliferative diabetic retinopathy
DPP	Diabetes Prevention Program
DN	Diabetic nephropathy
ESRD	End-stage renal disease
GFR	Glomerular filtration rate
DPN	Diabetic polyneuropathy
DAN	Diabetic autonomic neuropathy
CAN	Cardiac autonomic neuropathy
ANGPTL	Angiopoietin-like proteins
ANGPLT	Angiopoietin-like protein 3
ANGPLT4	Angiopoietin-like protein 4
ANGLPT8	Angiopoietin-like protein 8
LCN2	Lipocalin-2
MMP-9	Matrix metalloproteinase
NGAL	Neutrophil gelatinase-associated lipocalin
AGEs	Advanced Glycation End Products
CTRPs	C1q tumor necrosis factor-related protein
AMPK	AMP-activated protein kinase
MCP1	Monocyte chemoattractant protein 1
ICAM1	Intracellular adhesion molecule 1
APJ	Apelin receptor
Gal-3	Galectin-3
ALEs	Advanced lipoxidation end-products
SGLT2	Sodium–glucose cotransporter 2
NAFLD	Non-alcoholic fatty liver disease
GLP-1	Glucagon-like peptide-1
GLP-1 RAs	GLP-1 receptor agonists
GIP	Gastric inhibitory polypeptide
